# Carbon footprint of grain production in China

**DOI:** 10.1038/s41598-017-04182-x

**Published:** 2017-06-29

**Authors:** Dan Zhang, Jianbo Shen, Fusuo Zhang, Yu’e Li, Weifeng Zhang

**Affiliations:** 1 Centre for Resources, Environment and Food Security, China Agriculture University, Beijing, 100193 China; 2 Institute of Environment and Sustainable Development in Agriculture, Chinese Academy of Agricultural Sciences, Beijing, 100081 China

## Abstract

Due to the increasing environmental impact of food production, carbon footprint as an indicator can guide farmland management. This study established a method and estimated the carbon footprint of grain production in China based on life cycle analysis (LCA). The results showed that grain production has a high carbon footprint in 2013, i.e., 4052 kg ce/ha or 0.48 kg ce/kg for maize, 5455 kg ce/ha or 0.75 kg ce/kg for wheat and 11881 kg ce/ha or 1.60 kg ce/kg for rice. These footprints are higher than that of other countries, such as the United States, Canada and India. The most important factors governing carbon emissions were the application of nitrogen fertiliser (8–49%), straw burning (0–70%), energy consumption by machinery (6–40%), energy consumption for irrigation (0–44%) and CH_4_ emissions from rice paddies (15–73%). The most important carbon sequestration factors included returning of crop straw (41–90%), chemical nitrogen fertiliser application (10–59%) and no-till farming practices (0–10%). Different factors dominated in different crop systems in different regions. To identity site-specific key factors and take countermeasures could significantly lower carbon footprint, e.g., ban straw burning in northeast and south China, stopping continuous flooding irrigation in wheat and rice production system.

## Introduction

The carbon balance can be an indicator of agricultural production efficiency (net primary productivity is characterised by the capacity for CO_2_ fixation), soil fertility (soil organic carbon) and environmental pollution (global warming potential). So identifying the carbon footprint of a crop is an important component of sustainable agriculture^1, 2^. A carbon footprint of crop is defined as the carbon emission or carbon consequences induced by production practices in growing season^3–5^. Advances in carbon footprint research have supported carbon trade in 14 developed countries including the United States, Japan and various countries in the European Union^6^. For instance, carbon labelling is used to inform consumers of the greenhouse gas emissions generated throughout the entire life cycle of a product or service (i.e., its carbon footprint), which can help consumers select low-carbon products and guide producers towards low-carbon production strategies^7^. With the increase in carbon emissions and the need for social-economic transformation in China, a carbon market and associated management techniques are urgently needed^8, 9^, but there is insufficient research related to quantifying carbon footprints to support such a management system.

In recent years, grain production technologies, including the use of fertilisers and pesticides, have significantly changed in China, and the increase in agricultural inputs has been important to the development of sustainable agriculture as well as human society^10^. Not only do these inputs result in greenhouse gas emissions after their application in the field, but large quantities of greenhouse gases are generated during their upstream production. The development of agricultural machinery and increasingly sophisticated irrigation techniques has also contributed to high carbon emissions due to their reliance on fossil fuels. Straw burning has become an increasingly serious phenomenon as rural households have eliminated straw as a daily energy source, the practice not only produces significant greenhouse gas emissions but leads to air pollution and haze, both of which are harmful to human health. However, these changes have not been adequately considered in previous research^8, 11^, so there are uncertainties regarding their impacts that interfere with the development of low-carbon agriculture^12^.

Life cycle analysis (LCA) has been developed as a tool to estimate carbon emissions internationally^13^, but this methodology has not been applied to grain production in whole China. The existing research does not follow the “cradle-to-grave” principle of LCA, nor does it construct a site-specific context. For example, Cheng *et al*.^8, 14^ calculated the carbon footprint of grain production from national agricultural input data, but carbon emissions and carbon sequestration from agricultural processes such as fertiliser application, straw-returning, tillage and irrigation practices, were not considered. Other studies have only calculated carbon emissions from certain processes in the crop production chain. For example, Linquist *et al*.^15^ and Cole *et al*.^16^ estimated fertiliser-induced emissions of N_2_O from soil and CH_4_ from rice paddies but did not consider carbon emissions from the upstream production of agricultural inputs or fossil fuel consumption by agricultural machinery and irrigation. Some studies have included upstream and in-field emissions but failed to include all carbon emission sources. For instance, Chen *et al*.^9^ included carbon emissions from fertiliser production and nitrogen fertilisation, but they did not include emissions from manure, pesticides or agricultural films. Furthermore, farmland ecosystems are capable of both emitting and sequestering carbon, but while numerous studies have considered emissions, sequestration has been overlooked.

The establishment of a new method to calculate the carbon footprint of different crops, regions and production techniques is necessary to promote coordinated economic and environmental development. This study focused on matching local factors to production practices in the current farming system.

## Results

### Farming practices on three main grain crops in China

The survey results showed significant differences in the farming practices used in the eight major maize production zones in China as well as among farmers in the same zone (Table 1). In general, the N application rate exceeded 200 kg N/ha with a variation of 0–615 kg N/ha, and the N application rate in zones IV (the North China Plain) and III (lower basin of the Yangtze River (paddy-upland rotation area)) was much lower than in other regions due to the nutrient surplus from crop rotation. Nationally, only 16% of farmers applied manure for maize production, and no-till technologies were employed by 43% famers in IV, which was much greater than in other regions. In contrast to other regions, 94% of the spring maize in Northwest China was covered with mulch due to limited rainfall. Straw management also varied by region. Most straws in Northeast China were burnt due to the low temperature in winter and the lack of industrial applications, while 85% of the straws in IV were incorporated back into fields due to more moderate temperatures and high mechanisation. The electricity consumption for irrigation varied significantly among regions, being very high in IV due to the use of groundwater for flooding irrigation.

**Table 1:** Differences in the production techniques for three main crops across various primary production areas in China.

Crop	Region	Sample	Nitrogen fertiliser input	Diesel consumption	Electricity consumption for irrigation	Proportion of households applying manure	Proportion of households utilising no-till techniques	Proportion of households practicing straw returning	Proportion of households practicing straw burning
			kg N_2_O/ha	kg/ha	kW h/ha	%	%	%	%
Maize	I 101	50	253 ± 113 ab	326 ± 137 ab	127 ± 470 a	78 ± 42 c	12 ± 33 a	45 ± 48 b	31 ± 43 c
	I 103	40	232 ± 117 ab	317 ± 144 ab	159 ± 522 a	83 ± 38 c	18 ± 38 ab	46 ± 48 b	21 ± 37 c
	III 102	32	208 ± 79 a	518 ± 153 c	339 ± 460 ab	6 ± 25 a	0 ± 0 a	41 ± 42 b	17 ± 31 bc
	IV 102	703	214 ± 101 a	419 ± 195 bc	1888 ± 1262 c	4 ± 20 a	43 ± 49 b	78 ± 38 c	4 ± 15 ab
	V 101	16	328 ± 139 c	655 ± 210 d	1495 ± 2105 c	81 ± 40 c	0 ± 0 a	0 ± 0 a	0 ± 0 a
	VI 101	128	287 ± 118 bc	668 ± 220 d	1334 ± 3662 c	37 ± 48 b	0 ± 0 a	37 ± 47 b	3 ± 17 ab
	VI 102	85	246 ± 87 ab	500 ± 197 c	1205 ± 1486 bc	6 ± 24 a	21 ± 41 ab	84 ± 37 c	2 ± 11 a
	VII 101	195	231 ± 70 ab	292 ± 168 a	10 ± 83 a	14 ± 35 a	7 ± 26 a	1 ± 10 a	28 ± 42 c
Wheat	II 201	16	180 ± 48 a	379 ± 304 a	180 ± 347 a	19 ± 40 a	13 ± 34 b	19 ± 40 a	81 ± 40 c
	III 201	162	209 ± 71 ab	532 ± 183 b	14 ± 110 a	7 ± 25 a	1 ± 8 a	82 ± 37 b	11 ± 31 b
	IV 201	800	254 ± 102 b	635 ± 230 bc	3904 ± 3092 b	10 ± 30 a	0 ± 0 a	94 ± 23 b	2 ± 13 a
	VI 201	111	210 ± 83 ab	708 ± 222 c	1267 ± 2046 a	23 ± 42 a	0 ± 0 a	87 ± 33 b	1 ± 9 a
Rice	I 301	30	178 ± 44 b	417 ± 129 b	0 ± 0 a	37 ± 49 cd	0 ± 0	89 ± 30 bc	10 ± 30 ab
	I 302	104	185 ± 79 b	316 ± 203 a	16 ± 117 a	32 ± 47 cd	0 ± 0	80 ± 39 bc	19 ± 39 abc
	I 303	105	188 ± 84 b	320 ± 202 a	13 ± 114 a	24 ± 43 bc	0 ± 0	79 ± 40 bc	20 ± 40 abc
	II 301	142	154 ± 51 b	444 ± 181 bc	138 ± 540 a	50 ± 50 d	0 ± 0	72 ± 44 b	31 ± 46 bc
	II 302	88	154 ± 50 b	515 ± 145 cd	64 ± 200 a	25 ± 44 bc	0 ± 0	43 ± 49 a	56 ± 49 de
	II 303	91	156 ± 54 b	502 ± 142 bcd	61 ± 196 a	20 ± 40 abc	0 ± 0	45 ± 50 a	57 ± 49 de
	III 301	206	251 ± 108 c	549 ± 202 d	300 ± 958 a	8 ± 27 ab	0 ± 0	87 ± 32 bc	35 ± 47 cd
	IV 301	51	304 ± 102 d	586 ± 133 d	218 ± 545 a	20 ± 40 abc	0 ± 0	99 ± 0 c	0 ± 0 a
	VII 301	85	114 ± 35 a	766 ± 173 e	2157 ± 1074 b	1 ± 11 a	0 ± 0	29 ± 45 a	70 ± 45 e

Note: Mean ± SD. Different letters indicate significant differences among different regions of the same crop at P < 0.05 (ANOVA). Crop codes: 101 = spring maize; 102 = summer maize; 103 = autumn maize; 201 = winter wheat; 301 = single-crop rice; 302 = double-cropped early rice; 303 = double-cropped late rice. Area codes: I = triple-cropping area in South China, II = double-cropping rice area in South China, III = lower basin of the Yangtze River (paddy–upland rotation area), IV = North China Plain, V = irrigated area in Northwest China; VI = arid area in Northwest China, VII = single crop area in Northeast China.

The production practices used in the four wheat-producing areas of China differed markedly from one another as well as between households in the same area. The rate of nitrogen fertiliser application generally exceeded 200 kg N/ha, but that in IV was far greater than in other regions. In contrast, manure inputs were rare in most regions, except Gansu and Shaanxi (23% of the rural households used manure), while fuel consumption to operate machinery was generally higher than 600 kg/ha due to mechanisation throughout the entire production process. The proportion of straw returned to the field was especially high in region IV due to the high degree of mechanisation, moderate temperatures and the use of advanced technologies. In contrast, straw burning was significantly higher in Anhui and Jiangsu of II (double-cropping rice area in South China) because the wheat season is directly followed by the rice season. Therefore, straw is not able to decay before the next planting and thus would negatively affect rice production. Electricity consumption for wheat irrigation was generally higher in IV, primarily due to the dominance of well irrigation and the depth of the groundwater (Table 1).

Production practices also varied greatly across the 16 primary rice-producing areas of China as well as among farmers. The nitrogen fertiliser application rate exceeded 300 kg N/ha for single-crop rice fields in IV, and it exceeded 200 kg N/ha for single-crop rice in III. Manure application was rare in most regions with the exception of I and double-cropping rice areas. Fuel consumption for machinery operation was highest in single-crop rice fields in VII (Northeast China) because of the high degree of mechanisation. No-till techniques were not employed by any of the surveyed farmers (902 households in total). In VII, 70% of the straw was burned in the field due to the low temperature, but this proportion decreased to 45% in II and to 18% in I (triple-cropping area in South China) because the very tight rotation schedule resulted in the non-decomposed straw becoming hazardous for the subsequent rice crop. Significantly higher electricity consumption was found for single-crop rice fields in VII, which was likely due to the use of groundwater in this region while surface water or rainfall were the dominant water sources for rice production in the other regions.

### Carbon footprint of three grain crops in China

**Figure 1:**
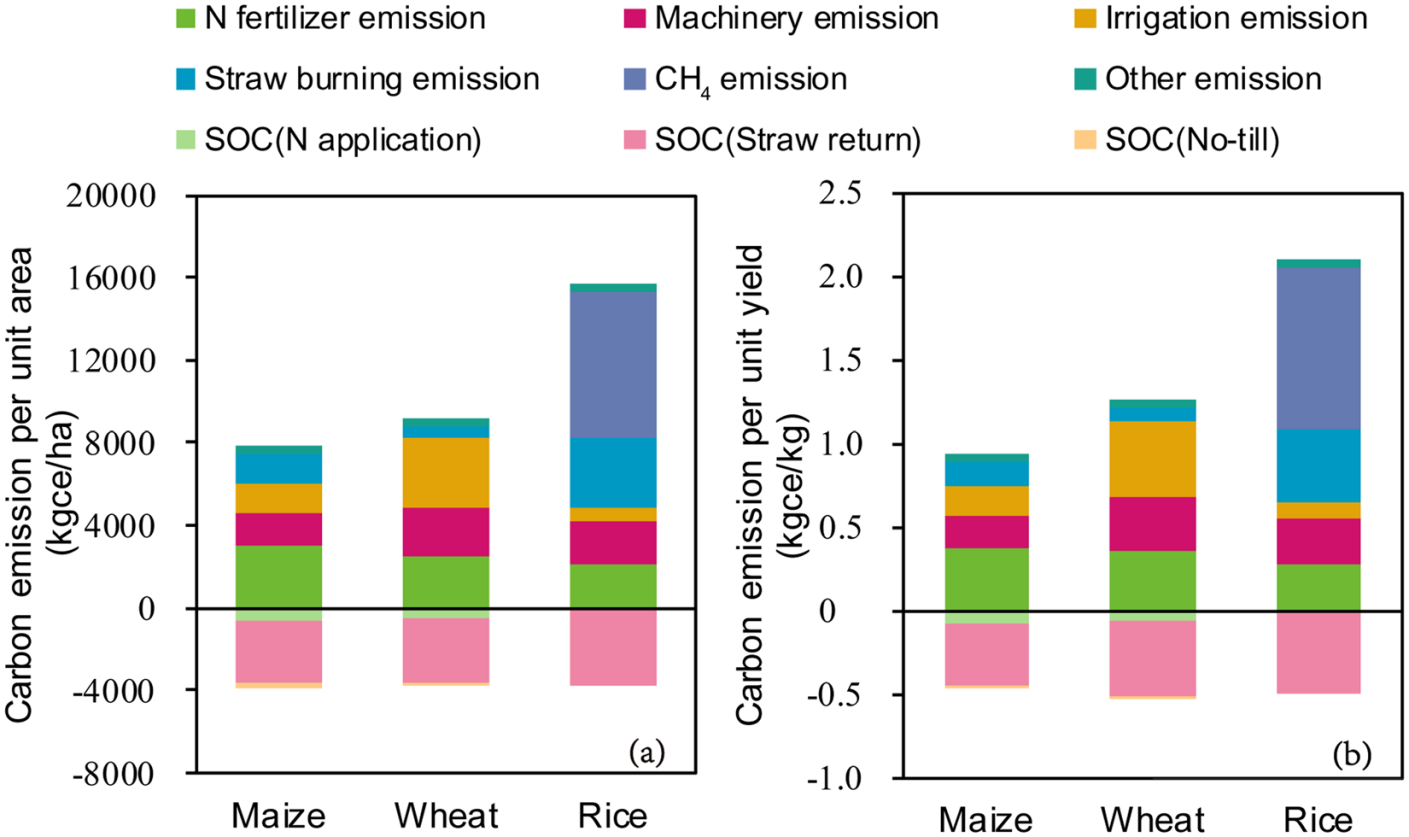
Carbon emission and carbon sequestration components per unit area/yield for the three primary grain crops in China. “Other emission” includes emissions from the upstream production and transport of agricultural inputs (such as chemical fertilisers, agricultural films, pesticides and manure).

We developed a carbon footprint inventory for maize, wheat and rice in different production regions based on the site-specific farming practices (Figs 1 and A4).

In calculating the carbon footprint (emission minus soil carbon sequestration), we found that the three crops emitted more carbon than they sequestered. Of the three main crops in 2013, maize had the lowest carbon footprint, i.e., 4052 kg ce/ha of carbon per unit area or 0.48 kg ce/kg per unit yield. The carbon footprint of wheat was 5455 kg ce/ha per unit area or 0.75 kg ce/kg per unit yield, while rice had the highest carbon footprint, i.e., 11881 kg ce/ha per unit area or 1.60 kg ce/kg per unit yield.

The factors contributing to these emissions varied markedly between crops. Rice yielded the greatest emissions (maximum: 15679 kg ce/ha) with 45% consisting of CH_4_ derived from paddy fields, 21% from straw burning, 14% from nitrogen fertiliser, 13% from fossil fuels for agricultural machinery and 4% from electricity consumption for irrigation. Wheat exhibited a high carbon emission value of 9119 kg ce/ha, of which 37% came from electricity consumption for irrigation, 28% from nitrogen fertilisers, 25% from fuel consumption by agricultural machinery and 6% from straw burning. Maize emitted 7900 kg ce/ha with 39% coming from nitrogen fertiliser, 20% from fuel consumption by agricultural machinery, 18% from electricity consumption for irrigation and 18% from straw burning.

There were also marked differences in the soil carbon sequestration under the three crops. In terms of carbon sequestration per unit area, maize ranked highest (3848 kg ce/ha) followed by rice (3798 kg ce/ha) and wheat (3664 kg ce/ha). For all three crops, carbon sequestration was primarily determined by straw treatment; for example, straw returning accounted for 96% of the carbon sequestration in rice paddies, while the remaining 4% was associated with the application of nitrogen fertiliser. Likewise, 80% of the carbon sequestration in maize fields resulted from straw returning while 15% was associated with the application of nitrogen fertiliser, and 5% stemmed from no-till practices. For wheat, 88% of the carbon sequestration was attributed to straw returning with the remaining 12% stemming from the application of nitrogen fertiliser.

### Carbon footprint of grains in different regions

#### Carbon footprint of maize in different regions

**Figure 2:**
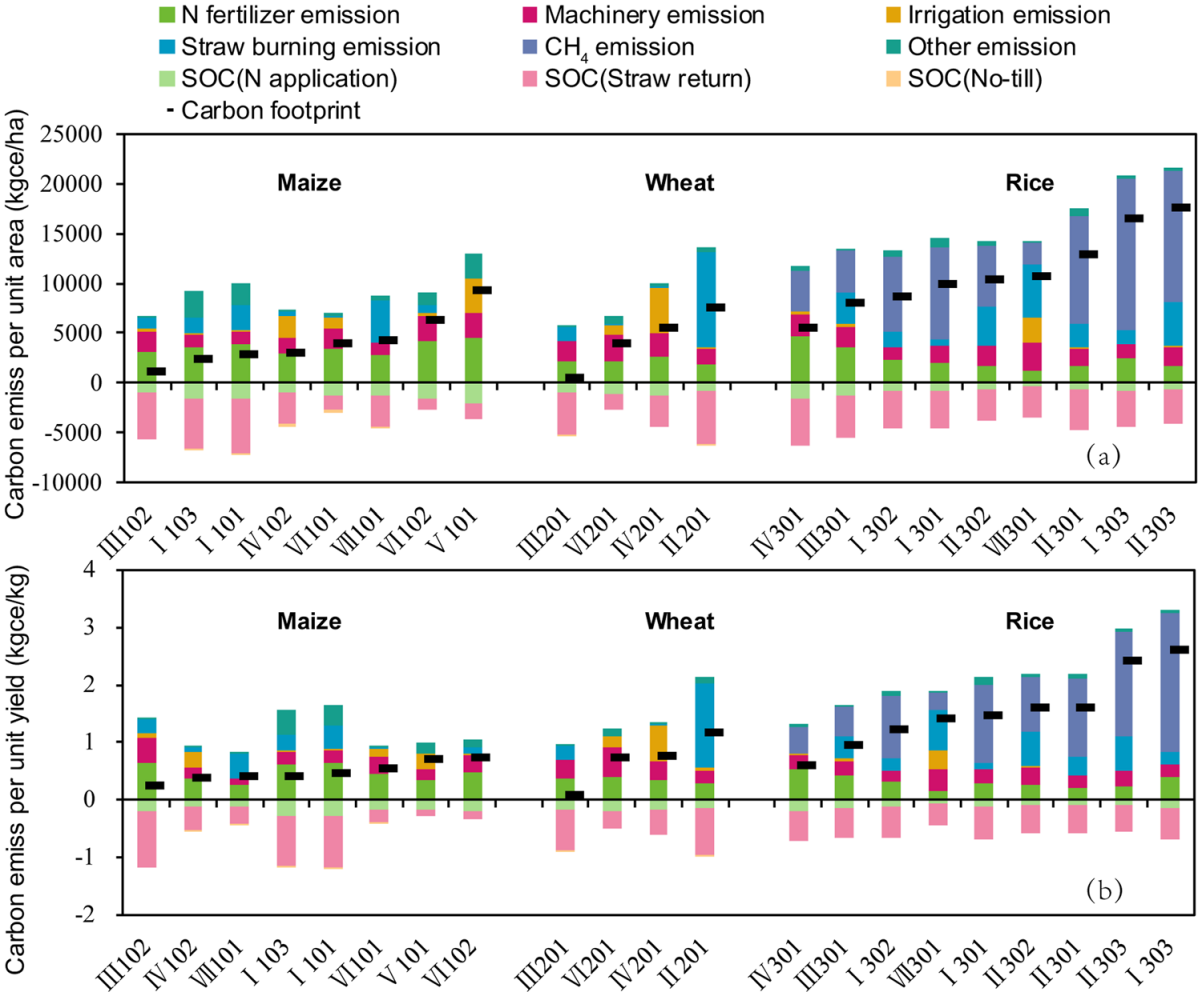
Distribution of carbon emissions and carbon sequestration among the three main crops in different areas. “Other emission” includes emissions from the upstream production and transport of agricultural inputs (such as chemical fertiliser, agricultural film, pesticides and manure). Crop codes: 101 = spring maize, 102 = summer maize, 103 = autumn maize, 201 = winter wheat, 301 = single-crop rice, 302 = double-cropped early rice, 303 = double-cropped late rice. Area codes: I = triple-cropping area in South China, II = double-cropping rice area in South China, III = lower basin of the Yangtze River (paddy-upland rotation area), IV = the North China Plain, V = irrigated area in Northwest China, VI = arid area in Northwest China, VII = single crop area in Northeast China.

The carbon footprint of maize production varied substantially among the eight regions in China, ranging from 1192 kg ce/ha to 9282 kg ce/ha (Figs 2a and A4) (the carbon footprint per unit yield ranged from 0.25 kg ce/kg to 0.73 kg ce/kg (Fig. 2b)) so that the regions could be divided into four groups. The greatest carbon footprint was found in V (irrigated area in Northwest China,the first group), where the carbon per unit area reached 9282 kg ce/ha. This result likely stemmed from the high overall carbon emissions (12967 kg ce/ha) and low carbon sequestration (3685 kg ce/ha), which can be explained as follows. (1) The general use of deep ploughing required fossil energy that resulted in high carbon emissions (46%), particularly when compared to no-till operations in northern China; in addition, electricity consumption for irrigation was relatively high. (2) Nitrogen fertiliser inputs were also much greater in this region, averaging 328 kg N/ha. (3) Low temperatures and drought conditions reduced the efficiency of soil organic carbon transformation after straw returning. For instance, carbon sequestration associated with straw returning reached 44 kg C/t straw in I, while it was only 17 kg C/t straw in V.

The second group included summer maize in VI (arid area in Northwest China), where the carbon per unit area reached 6276 kg ce/ha; this area also had the lowest carbon sequestration values (2762 kg ce/ha) and relatively high carbon emissions per unit area (9038 kg ce/ha). The carbon footprint of summer maize in the second group was similar to that in the first group and explained by similar factors, namely, high nitrogen fertiliser inputs, high fossil fuel consumption by agricultural machinery and low soil carbon sequestration efficiency. The primary difference between these two groups was that VI was rain-fed, so emissions from energy consumption for irrigation were zero.

The third group included spring maize in VI, spring maize in VII, spring and autumn maize in I and summer maize in IV. Despite the high carbon sequestration values recorded in these areas, the carbon emissions per unit area were relatively high, which was partly due to straw burning. For example, grain yield and straw production were high for spring maize in VII, while straw decomposition was slow due to low temperatures. Meanwhile, there was a lack of industrial demand for straw, which resulted in straw burning-induced carbon emissions of 4331 kg ce/ha. In region I, straw burning was relatively popular because straw returning negatively affected rice cultivation^17^, resulting in carbon emissions of 2557 kg ce/ha and 1575 kg ce/ha for the two crop types, respectively. However, the high carbon emissions were also the result of a high degree of mechanisation (i.e., film mulching in VI) that resulted in significant emissions from fossil fuel consumption.

The lowest maize carbon footprint was found for summer maize in III (1192 kg ce/ha), where there were low carbon emissions per unit area (6796 kg ce/ha) and a relatively high degree of carbon sequestration per unit area (5604 kg ce/ha). In this area, straw burning was not prominent; emissions from energy consumption for irrigation were not high; and the carbon sequestration efficiency was relatively high.

**Figure 3:**
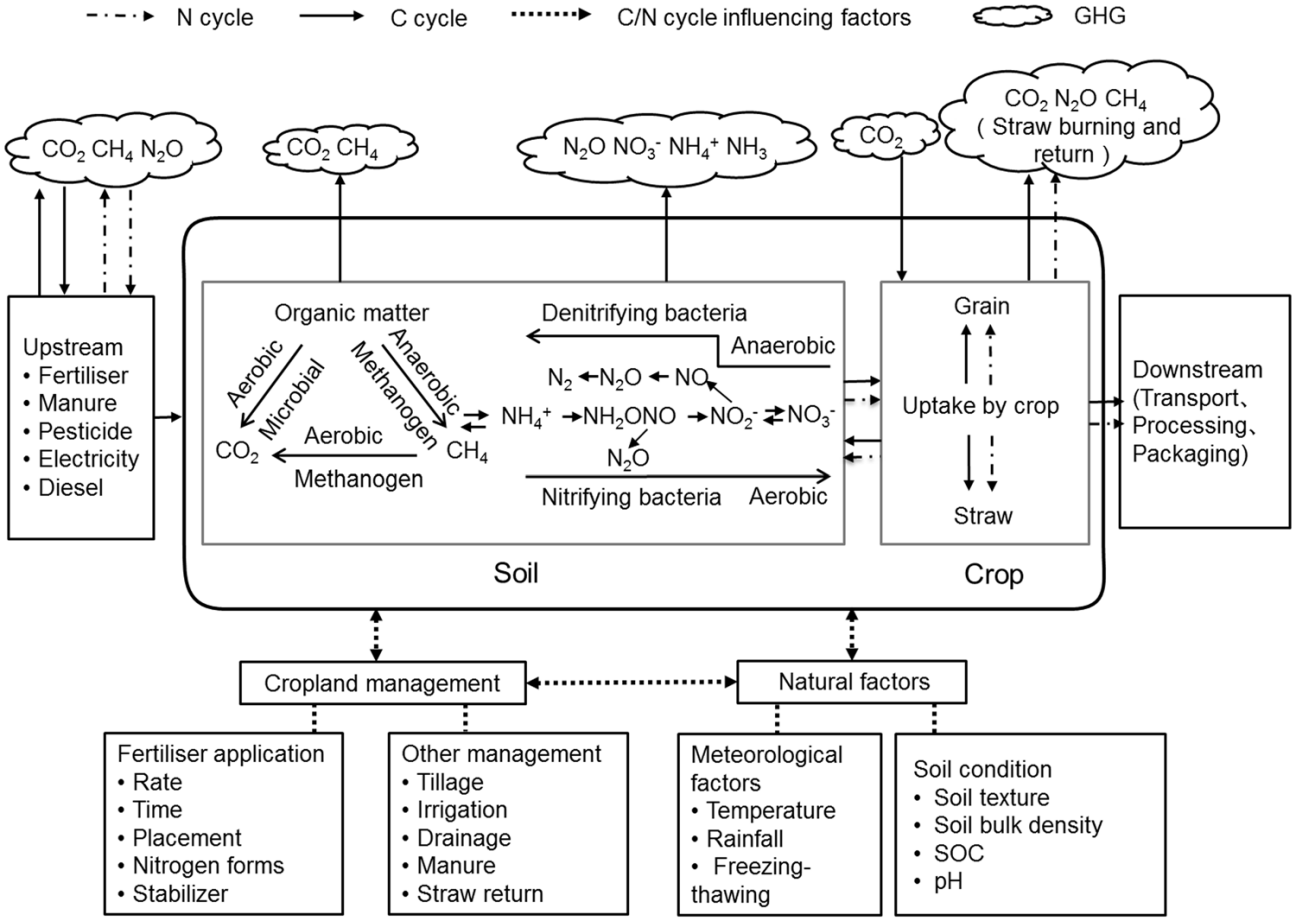
A theoretical model of the carbon footprint of crop production^6^.

The grain yields per unit area were not linearly correlated with the carbon footprint per unit area, so the carbon footprints per unit grain yield showed slightly different trends (Fig. 2b). For example, the carbon footprint in VII (4340 kg ce/ha in Fig. 3a) was higher than that in I (2885 kg ce/ha in Fig. 3a), but the grain yield per unit area in VII (10393 kg/ha) was also higher than that of summer maize in I (6059 kg/ha). Consequently, the carbon footprint per kg grain in the former region (0.41 in Fig. 3b) was lower than that in the latter (0.48 in Fig. 3b).

#### Carbon footprint of wheat in different regions

The total carbon emissions differed markedly across the four wheat production areas of China. The carbon footprint per unit area ranged from 502 kg ce/ha to 7513 kg ce/ha (Figs 2a and A4) (per unit yield ranged from 0.08 kg ce/kg to 1.17 kg ce/kg (Fig. 2b)) and was highest for winter wheat in II (7513 kg ce/ha). Although this area had the highest carbon sequestration values among the studied areas (6163 kg ce/ha), its total carbon emissions were also the highest (13676 kg ce/ha). This result was likely due to the high straw burning ratio in this area, which reached 81% and contributed 70% of the emissions.

Winter wheat in IV ranked second (5619 kg ce/ha), with carbon emissions per unit area of 10042 kg ce/ha and carbon sequestration of 4423 kg ce/ha, for the following reasons. (1) The reliance on deep groundwater for irrigation resulted in the attribution of 44% of the total emissions to electricity consumption. (2) Nitrogen fertiliser inputs were high, i.e., 254 kg N/ha, so carbon emissions accounted for 27% of the total emissions. (3) Mechanisation was also high, and the related fossil fuel consumption accounted for 24% of the total emissions.

Winter wheat in VI ranked third (3999 kg ce/ha), and this area exhibited the lowest carbon sequestration levels per unit area (2651 kg ce/ha). However, the carbon emissions per unit area were not high (6650 kg ce/ha) because low temperatures and drought conditions reduced the efficiency of soil organic carbon transformation after straw returning compared to the other areas (17 kg C/t, as discussed above).

Winter wheat in III ranked last in terms of the carbon footprint per unit area (502 kg ce/ha) due to the high soil carbon sequestration efficiency (as analysed above) as well as the low carbon emissions per unit area (5781 kg ce/ha). Carbon emissions were lower because straw burning was uncommon (11% in Table 1); the electricity consumption for irrigation was low (14 kW h/ha in Table 1); and energy consumption for machinery was minimal.

#### Carbon footprint of rice in different regions

The carbon footprint differed substantially between the nine rice production areas in China, ranging from 5502 kg ce/ha to 17568 kg ce/ha (Figs 2a and A4) (the per unit yield ranged from 0.62 kg ce/kg to 2.62 kg ce/kg (Fig. 2b)). The nine study regions can be divided into the following three groups: (1) late rice in II and late rice in I; (2) single rice in II, early rice in II, single rice in I, single rice in VII, early rice in I and single rice in III; and (3) single rice in IV.

Regions in the first group exhibited the highest carbon footprint per unit area (>15000 kg ce/ha) primarily due to high emissions, i.e., 17568 kg ce/ha and 16542 kg ce/ha for late rice in II and I, respectively, which were attributed to high CH_4_ output that accounted for 61–73% of total emissions. Among the households, 23% and 20% were found to apply manure and continuous irrigation, respectively, which resulted in CH_4_ emission factors of up to 32.2 mg CH_4_/(m^2^ h) and 22 mg CH_4_/(m^2^ h), respectively. These values were markedly higher than those found for the other areas. In addition, straw burning was a problem to be solved for late rice in II, accounting for 20% of emissions.

The high carbon emissions in the second group were due to the following reasons. (1) Except for single-cropping rice in VII, the proportion of emissions stemming from CH_4_ outputs was relatively high (32–63%). (2) Emissions from straw burning constituted 38% of the total emissions for single-crop rice in VII. (3) Emissions from nitrogen fertiliser reached 27% for single-crop rice in the paddy-upland rotation area. (4) High emissions from the operation of machinery and energy consumption for irrigation accounted for 20% and 17%, respectively, of the total carbon emissions from single-crop rice in VII.

The third group exhibited the lowest carbon emissions per unit area (11807 kg ce/ha) and the highest carbon sequestration per unit area (6305 kg ce/ha) for the following reasons. (1) The CH_4_ emission factor of rice paddies in the north was lower than that in the south due to climate and soil factors. On the other hand, the ratio of households who used intermittent irrigation without manure application was relatively high in this area (maximum: 46%). (2) These groups exhibited the highest soil carbon sequestration from straw returning and nitrogen fertiliser application (high mechanisation with 99% returned straw); carbon sequestration through the application of nitrogen fertiliser was linearly and positively correlated with the application rate. Although the carbon sequestration efficiency of nitrogen fertiliser in this area was low (0.53 kg C/kg N) compared to VI (1.74 kg C/kg N) and the southern areas (I, II and III) (1.53 kg C/kg N), nitrogen fertiliser was applied at much higher rates (304 kg N/ha) compared to the south (196 kg N/ha).

The yield levels per unit area were not linearly correlated with the carbon emission levels of the study areas.

## Discussion

### Carbon footprint of grain crops in China

The carbon footprints (per unit area and per unit grain yield) obtained in this study is different than those of other studies. For example, the carbon footprint of wheat in our research is 5455 kg ce/ha, which is higher than 794 kg ce/ha in Cheng *et al*.^18^ and 3000 kg ce/ha in Yan *et al*.^19^ (Table 2). That mainly due to different system boundaries, the data sources for emission factors and farming practices.

**Table 2:** Carbon footprint per unit area and carbon footprint per unit yield from different studies of grain crop production in China.

	Sample size	Yield	Carbon footprint per unit area	Carbon footprint per unit yield	Calculation method	Data source	Reference
		kg	kg ce/ha	kg ce/kg			
Maize	—	6791	781	0.12	A	National statistics	Cheng *et al*.^18^
	58	7000 ± 200	2300 ± 100	0.33 ± 0.02	A	Household survey	Yan *et al*.^19^
	1249	8480	4052	0.48	B	Household survey	This study
Wheat	—	5550	794	0.14	A	National statistics	Cheng *et al*.^18^
	48	4800 ± 200	3000 ± 200	0.66 ± 0.03	A	Household survey	Yan *et al*.^19^
	1089	7548	5455	0.75	B	Household survey	This study
Rice	—	6716	2472	0.37	A	National statistics	Cheng *et al*.^18^
	17	7600 ± 100	6000 ± 100	0.80 ± 0.02	A	Household survey	Yan *et al*.^19^
	902	14123	11881	1.60	B	Household survey	This study

Note: “A” indicates the upstream emission+ field emission (no manure), and “B” indicates the upstream emission+ field emission+ straw-burning emission after harvest.

Although Yan *et al*.^19^ used farmer survey data, it was limited by sample size and thus do not adequately represent potential regional variations (i.e., only 58 samples reported by Yan *et al*.^19^). In this study, 3240 farmer survey data across seven grain production areas was used, which can clearly reflect the farmers real practices and regional variations. For example, the carbon footprint of winter wheat in Yan’s study is 3000 kg ce/ha, which is similar to our study in Henan province (3792 kg ce/ha) in region IV, because his study region for winter wheat is Henan. In fact, regions IV comprise 9 provinces (Henan, Shandong, Hebei, Anhui, Jiangsu, Beijing, Tianjin, Gansu, Shannxi) and the variation of carbon emission in region IV is very high (2397 kg ce/ha-30825 kg ce/ha), because farmers’ practices (i.e. fertiliser and irrigation) is differed markedly from one another.

In present study, the carbon footprint included emissions from field emissions and upstream inputs (production and transportation of fertiliser, electricity, chemicals and machinery), thus accounting for the complete grain production life cycle. The emissions from upstream inputs account for 63%, 86% and 50% total emissions of maize, wheat and rice respectively. Yan’s study^19^ had typically adopted “half” life cycle analysis approaches and ignored emissions from manure processing and application, straw burning, and plastic film. These ignored emissions account for 2842 kg ce/ha, 1226 kg ce/ha and 3873 kg ce/ha of maize, wheat and rice respectively.

Most of studies used the national statistical data (such as Cheng *et al*.^18^). The statistical data supply general agriculture inputs, but cannot reflect critical regional farming practices, such as straw treatment and manure processing and application, which are critical factors for carbon footprints. For example, the straw burning was responsible for an average of 18% (as high as 70% for wheat in II), and manure processing and application was responsible for an average of 15% (as high as 70% for autumn maize in I). Therefore, the ignored emissions from manure and straw burning (i.e. ref. 18) underestimated carbon emission by 2825 kg ce/ha, 1226 kg ce/ha and 3873 kg ce/ha for maize, wheat and rice respectively.

Furthermore, this study accounted for soil carbon sequestration while other two studies did not. But soil carbon sequestration is an important component of the field-based carbon footprint. The organic carbon pool has been changing dramatically throughout the arable soils of China^20, 21^, to build up of the soil organic carbon pool is an important target of sustainable development. In other countries, soil carbon sequestration were included in the carbon footprint (i.e. 1100 kg ce/ha soil carbon sequestration in no-till maize in ref. 22).

When compared to other countries, the carbon footprint per unit yield of maize, wheat and rice in China were noticeably higher than those in the United States, Canada and India (Table 3). They adopted similar system boundary as we are, i.e. they calculated the net emissions including soil carbon sequestration, field emissions, and upstream emission from N fertiliser, lime, fuel (excluded P fertiliser, K fertiliser, plastic film, manure, and straw burning). The big gap between China and other countries mainly ascribe to different emission factors and farmers practices, which are all related to management technologies. For example, the emission factor for N manufacture and transport is 8.31 kg ce/kg N in China, comparable 4.51 kg ce/kg N in USA. The N application rate is 230 kg N/ha for maize, compared to 67 kg N/ha in the research region of USA^22^ (the average N application rate in USA national scale is 152 kg N/ha^23^).

**Table 3:** Comparison of carbon emissions from China and other countries.

Crop	Country	Carbon emissions per unit yield	Contribution of nitrogen fertiliser	Reference
		kg ce/kg		
Maize	United States	0.12–0.25	45–75%	Snyder *et al*.^22^
	China	0.95 (0.41–0.85)	40% (32–49%)	This study
Wheat	United States	0.25–0.35	67–75%	Snyder *et al*.^22^
	India	0.12	75%	Pathak *et al*.^66^
	Canada	0.27–0.50	—	Gan *et al*.^67^
	China	1.26 (0.21–1.30)	28% (13–33%)	This study
Rice	India	1.2–1.5	<10%	Pathak *et al*.^66^
	China	2.10 (0.72–2.74)	15% (8–40%)	This study

Therefore, our results implied that the crop production practices in China have big potential to be optimized. For example, excess nitrogen fertiliser is currently being applied on each crop in national scale. The best nutrient management practices (right amount, right products and right application method) could lower carbon footprint significantly. Moreover, the proportion of emissions from electricity consumption for irrigation was higher for wheat, which due to the use of flood irrigation. Water-saving irrigation techniques could be applied to minimise these carbon emissions. The proportion of emissions from straw burning was tremendous for rice and maize, especially in the northeast, so optimising the energy associated with straw regeneration and straw decomposition technologies is critical for reducing carbon emissions in this region. Additionally, CH_4_ emissions were also enormous for rice, but intermittent irrigation can be enhanced as a form of mitigation, especially for late rice in II and I, where CH_4_ emission factors are high.

### Perspectives on the carbon footprint estimation method

Establishing a complete life cycle approach for carbon footprint estimation requires ever-evolving systematic research; in particular, more detailed site-specific parameters are required to understand the interactions among parameters. For example, we found the following uncertainties in the N_2_O emission factor in the present study. (1) The N_2_O emission factor is only able to simulate the rate of nitrogen fertiliser application (or nitrogen surplus); it does not reflect the influence of climate and soil factors^24–26^ and does not fully capture regional differences. (2) Interactions between different factors may affect the results; for example, the condition of soil water has a major impact on the emission of N_2_O from rice paddies. Additionally, there is a reciprocal relationship between the carbon emissions from CH_4_ and N_2_O^27, 28^, while straw returning affects N_2_O emissions to a certain extent by altering nitrification and denitrification processes^29–32^. To date, research has failed to find consistent solutions to these issues^33^. (3) Manure-induced carbon emissions have a great impact on the overall carbon footprint, but there are large differences in the types and treatment of manure, which substantially alter the parameters of interest. In this study, our parameters were based on data from research on pig dung, which accounts for 48.2% of the total livestock excrement in China^34^. However, the application of cow dung is common in many areas, particularly in the north and northwest^34^. Furthermore, production processes associated with animal faeces and urine affect the nitrogen content of manure to a certain extent, thus influencing the emission of N_2_O and CH_4_^35^. These examples indicate that the current parameters used to estimate carbon footprints are insufficiently detailed.

## Conclusions

When the complete life cycles of the three primary grain crops produced in China were considered, the carbon emissions from the production of these crops were found to be much higher than the associated soil carbon sequestration, suggesting that they are net emission systems. High water and fertiliser inputs as well as the operation of machinery for agricultural purposes not only induced soil N_2_O and CH_4_ emissions but resulted in considerable consumption of energy from fossil fuels. Straw burning further increased the carbon emissions of these grain crops. It is recommended that intermittent irrigation be optimised to reduce the emission of CH_4_ from rice paddies and that the irrigation of wheat, nitrogen fertiliser management for maize and straw returning for rice and maize be optimised to reduce the carbon footprint of these crops.

### Study methods

The carbon emissions from grain crop production consist of the following four components: (1) emissions from the upstream production and transport of agricultural inputs, (2) direct or indirect emissions induced by various agricultural management processes, (3) emissions from the burning of crop residues and (4) the soil carbon sequestration associated with various production methods (i.e., minimum and no-till practices, straw returning and fertilisation) implemented during crop growth.

According to LCA theory, the field production carbon footprint includes carbon emissions and carbon sequestration at two points in the production life cycle: the upstream links are associated with the creation of inputs used in agricultural production and processing (i.e., seeds, fertiliser, pesticides, machinery and agricultural film) and the in-field emissions and sequestration are associated with field cultivation (i.e., tillage, sowing, plant protection, harvesting and straw treatment) (Fig. 3).

In the above model (Fig. 3), the emission types include greenhouse gases (CO_2_, N_2_O and CH_4_), and sequestered carbon refers to the organic carbon in the soil (Table 4). Therefore, various types of emissions need to be calculated at different points in the crop production cycle. Meanwhile, natural factors, such as climate and soil, play an important role in the microbial mediation of greenhouse gas emissions through processes such as nitrification and denitrification, CH_4_ emission from rice paddies and soil carbon sequestration. These conditions differ markedly between various primary production areas, so regional differences must be taken into consideration when calculating carbon emission and sequestration components.

**Table 4:** Carbon forms in the carbon footprint model.

Path	Factor	Carbon form	Differences in emission and sequence factors among regions
Emissions from agricultural inputs, production and transportation	Chemical fertiliser (N, P, K)	CO_2_	—
	Manure	N_2_O, CH_4_	—
	Pesticide	CO_2_	—
	Film	CO_2_	—
	Diesel	CO_2_	—
	Electricity	CO_2_	—
Field emissions of N_2_O	Nitrogen fertiliser application	N_2_O	—
	Manure application	N_2_O	—
Emission of CH_4_ from paddy fields	Rice plant and paddy field	CH_4_	√
Carbon sequence	Nitrogen fertiliser application	SOC	√
	Straw returning	SOC	√
	No-till	SOC	√
Emission from straw burning	Straw burning	CO_2_, N_2_O	—

### Formulae used to determine the carbon footprint of a grain crop production system

$${{\rm{nGWP}}}_{{\rm{CO2}}}={{\rm{GWP}}}_{{\rm{CO2}}}-{\rm{SOCSR}}$$

GWP_CO2_ is the carbon emissions from grain crop production per unit area (kg ce/ha), SOCSR is the carbon sequestered per unit area by grain crop production (kg ce/ha). More details are available in the supplementary (Appendix B).

### Factor selection for the carbon footprint model

#### Soil N_2_O emission factor

According to the “Study on the Greenhouse Gas Inventories in China”^36^, three types of nitrogen sources yield direct N_2_O emissions from crop cultivation: chemical nitrogen fertiliser applied in-field, manure applied in-field and crop straw inputs returned directly to the field, which retain residual nitrogen in the root stubble. In addition, three primary nitrogen sources result in indirect N_2_O emissions from crop cultivation: atmospheric nitrogen deposition due to ammonia volatilisation and nitrogen oxide emission from fertilised fields, nitrogen discharged into water bodies through leaching or runoff from manure-treated fields and atmospheric nitrogen deposition from nitrogen-containing active substances released from crop straw during field burning (calculated as part of straw burning).

In this study, the N_2_O emissions model described in Chen *et al*.^9^ was selected to represent the N_2_O emission factor for the application of chemical nitrogen fertiliser. This model is based on studies of nitrogen loss, including ammonia volatilisation, nitrate leaching and N_2_O emissions in the primary grain-producing areas of China before June 2012; it was published in *Nature*.

The N_2_O emission factor for manure application was synthesised from the literature on field manure management in China and overseas prior to the beginning of 2014A total of 62 samples were obtained from 10 studies of direct N_2_O emissions^37–46^, and 211 samples were derived from 13 studies related to NH_3_ volatilisation-induced indirect N_2_O emissions^37, 40, 42, 47–56^. More details are available in the supplementary (Appendix C1).

#### Rice paddy CH_4_ carbon emission factor

Only CH_4_ emissions from rice paddies were considered in this study, and there were large differences due to a series of factors including soil type, climate conditions, rice variety, irrigation method and field management. To reflect these differences, we used the CH_4_-related carbon emission model created by Yan *et al*.^57^ for rice paddies in China based on 204 records collected from 23 sampling points. The independent variables included four categorical variables: rice-growing area (five rice-producing areas), manure application (with or without), watering method (intermittent irrigation or continuous flooding) and rice type (early, middle or late).

#### Soil SOC sequestration factor

In this study, we used the empirical model for soil carbon sequestration proposed by Lu *et al*.^58^. The database linked to this model contains data from long-term, fixed-point experiments performed in the primary agricultural production areas of China; the large sample selected for this study was collected over more than three years and included results from 93 studies and 220 data pairs. More details are available in the supplementary. The soil carbon sequestration was calculated based on the specific farmers practices (straw treatment, reduce tillage, fertilizer input), and the SOC conversion factors (using the empirical model from ref. 58) (Appendix C2).

#### Straw-burning carbon emission factor

The carbon emission factor for straw burning used in this study was derived from the results discussed in Zhang *et al*.^12^, who used a custom-designed combustion and test device to simulate the open burning of straw in fields. More details are available in the supplementary (Appendix C3).

#### Carbon emission factor of upstream production and transport of agricultural inputs and the carbon emission factor of manure

According to LCA theory, carbon emissions produced during the upstream development of agricultural inputs required for grain crop production should be considered when determining the carbon footprint of a system. These production goods primarily include nitrogen fertiliser, phosphate fertiliser, potassium fertiliser, pesticides, agricultural film, agricultural machinery and manure composting. Moreover, modern agricultural production processes consume a large amount of energy in the form of the diesel fuel and electricity required to operate machinery and facilitate irrigation.

Based on the LCA method, Zhang *et al*.^59^ quantified greenhouse gas emissions from the production and transport of nitrogen fertiliser as well as the production processes, energy types and energy consumption efficiency of 230 nitrogen fertiliser manufacturers nationwide. In that study, the emission of greenhouse gases in the production of nitrogen fertiliser reached 6.9 tons of CO_2_ per ton of fertiliser; this factor was published in *PNAS*. Additionally, Chen *et al*.^60^ analysed data from China and used the LCA method to calculate carbon emission factors at the general manufacturing level for phosphate and potassium fertilisers.

In the present study, the model assumes that all manure inputs are pig dung, and the carbon emission factors for sludge composting and pig dung composting (fossil carbon emissions from transport, machinery operation, processing and drying and CH_4_ and N_2_O emissions from composting) were 177.75 and 189.14 kgce t^−1^ DS^61^, respectively. Based on nitrogen content values of 2.18% and 1.36%, the average carbon emission factor of manure (2% for the total nitrogen content; C:N = 13:1) was estimated to be 0.223 kg ce/kg dry matter^62^.

Modern pesticides are produced using fossil fuel-based energy^63^, and various types of pesticides have different carbon emission levels per unit area/yield^64^. As pesticide production is often regarded as a trade secret in China, most of the data related to energy consumption has been published overseas. In the present study, we used the carbon emission factor determined by Audsley *et al*.^65^, which includes the production, distribution, packaging and transport of pesticides. Formula fitting was based on pesticide composition.

During the collection of survey data from rural households in the present study, farmers had difficulty accurately describing the components of specific pesticides due to the complexity of their composition; in addition, most packing bags were discarded after use and were unavailable for review. Therefore, only the dosages of pesticides, fungicides and herbicides were investigated in the questionnaire, and specific components were not analysed. The carbon emission parameters of three types of pesticide (i.e., herbicides, pesticides and fungicides) were selected following the method in Audsley.

### Crop division method

In this study, we divided the main grain crop-producing areas of China by climatic zone and cropping system. First, China was divided into the following three main areas by the annual cumulative temperature: a tropical/subtropical zone, a warm temperate zone and a middle temperate zone. These areas were further divided into the following seven subareas: I (triple-cropping area in South China), II (double-cropping rice area in South China), III (lower basin of the Yangtze River (paddy–upland rotation area)), IV (North China Plain), V (irrigated area in Northwest China), VI (arid area in Northwest China) and VII (single crop area in Northeast China). More details are available in the supplementary (Appendix D). A rural household survey of the typical crops in each area was carried out in 2014, and the following three main grain crops were determined: wheat, maize and rice. The survey samples included 3240 households from 42 counties in 11 provinces (regions), including Heilongjiang (including the reclamation area), Jilin, Hebei, Henan, Shandong, Shaanxi, Gansu, Anhui, Jiangsu, Hunan and Guangxi.

### Data sources

The indicators assess in the survey questionnaire included the maximum plot yield, maximum plot area and growth period of crops; the rate of application of chemical fertiliser, manure, pesticides and film; the fuel consumed in the operation of machinery (soil preparation, sowing, fertilisation and harvesting) and the electricity consumed for irrigation; the tillage (ploughing and minimum or no-till) and rice irrigation (intermittent irrigation or long-term flooding) practices; and the amount of straw burning and straw returning. More details are available in the supplementary (Appendix E).

## Electronic supplementary material

Supplementary information
