# Supplementary material for: Carbon footprint of grain production in China
Source: Sci Rep. 2017 Jun 29;7:4126. doi: 10.1038/s41598-017-04182-x (PMC5491493; doi:10.1038/s41598-017-04182-x)
Supplement: Supplementary file 1 — Supplementary information [file 41598_2017_4182_MOESM1_ESM.doc]

**Carbon footprint of grain production in China**

**Dan Zhang^1^, Jianbo Shen^1^, Fusuo Zhang^1^, Yu’e Li^2^, Weifeng Zhang^1^**

^1^ Centre for Resources, Environment and Food Security, China Agriculture University, Beijing 100193, China

^2^Institute of Environment and Sustainable Development in Agriculture, Chinese Academy of Agricultural Sciences, Beijing 100081, China

Correspondence to [wfzhang@cau.edu.cn](mailto:wfzhang@cau.edu.cn)

**Supplementary information**

**Appendix A *The distribution of the sampled maize, wheat and rice***

Figure A1 The distribution of the sampled maize

Figure A2 The distribution of the sampled wheat

Figure A3 The distribution of the sampled rice

Figure A4 The distribution of the carbon footprint of maize, wheat and rice in China

**Appendix B *Formulae used to determine the carbon footprint of a grain crop production system***

**Appendix C *Factor selection for the carbon footprint model***

**Appendix D *Crop division method***

**Appendix E *Date source***

**Appendix A** The distribution of the sampled maize, wheat and rice


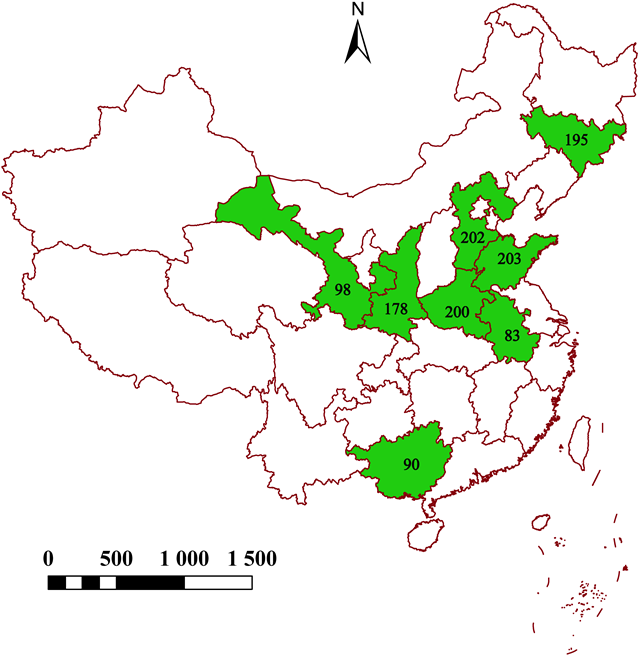


Figure A1 The distribution of the sampled maize. The map created by the ArcGIS 10.2 software package. <http://www.esri.com/software/arcgis>


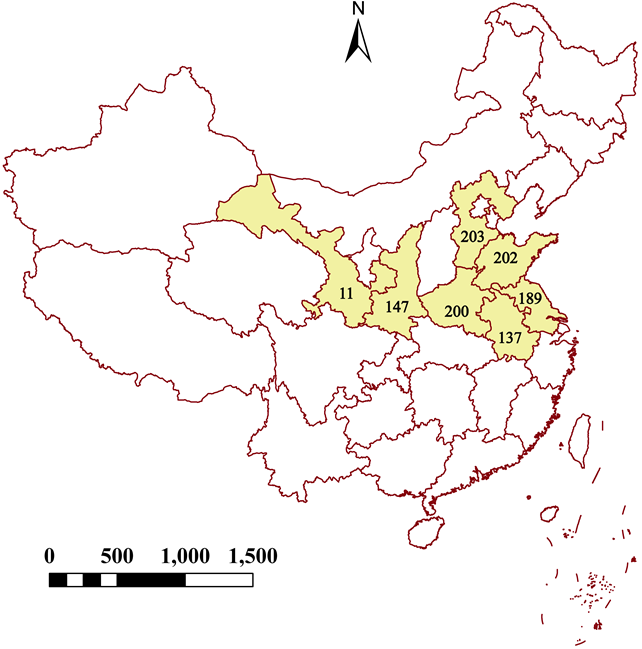


Figure A2 The distribution of the sampled wheat. The map created by the ArcGIS 10.2 software package. <http://www.esri.com/software/arcgis>

**
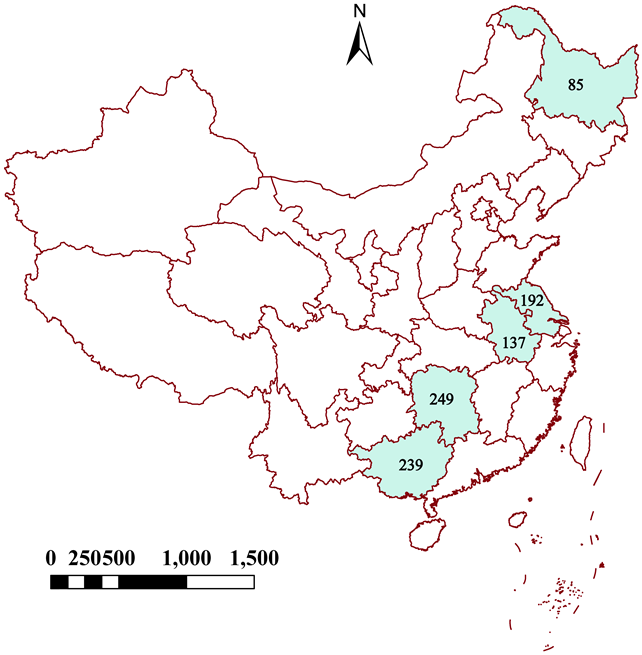
**

Figure A3 The distribution of the sampled rice. The map created by the ArcGIS 10.2 software package. <http://www.esri.com/software/arcgis>

**
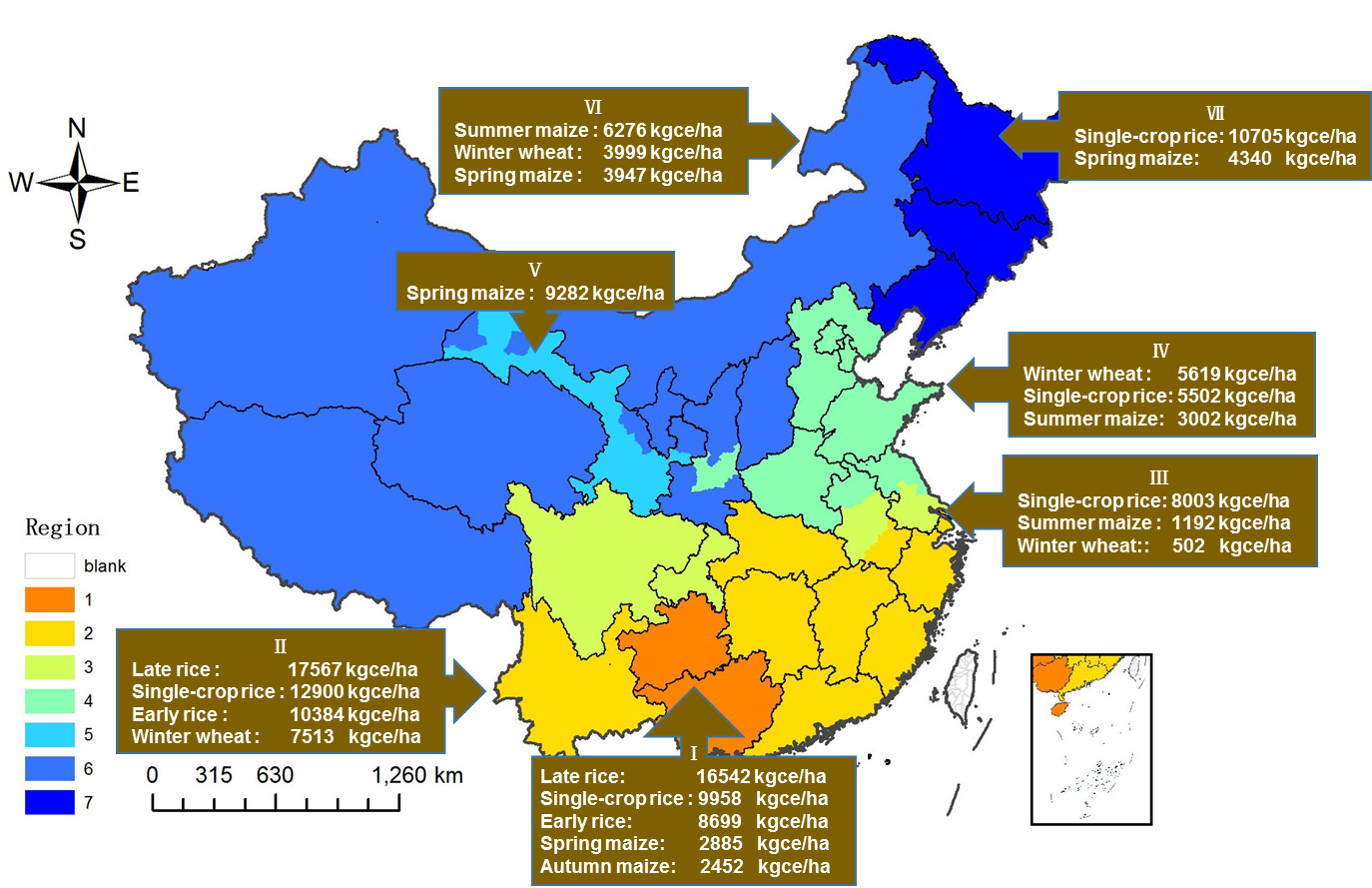
**

Figure A4 The distribution of the carbon footprint of maize, wheat and rice in China. The map created by the ArcGIS 10.2 software package. <http://www.esri.com/software/arcgis>

**Appendix B *Formulae used to determine the carbon footprint of a grain crop production system***

(1) Carbon emissions formula

GWP_CO2_=CO_2input_+N_2_O_soil_×298+CH_4paddy_×25 (1)

CO_2input_=Fer_N_×EF_FerN_+Fer_P_×EF_FerP_+Fer_K_×EF_FerK_+Manu×EF_Manu_+Pest×EF_Pest_+Film×EF_Film_+Dies×EF_Dies_+Elec×EF_Elec_+Straw×EF_Burning_ (2)

N_2_O_soil_=Fer_N_×EF_FerN-N2O_+Manu×EF_Manu-N2O_ (3)

CH_4paddy_=EF_CH4_×Growth-Days (4)

(2) Carbon sequestration formula

SOCSR=(C_soc-FerN_+C_soc-straw_+C_soc-notil_)×44/12 (5)

C_soc-FerN_ =Fer_N_×SF_FerN_ (6)

C_soc-straw_=Straw×SF_Straw_ (7)

C_soc-notil_=Area-notil×SF_notil_ (8)

(3) Carbon footprint formula

nGWP_CO2_= GWP_CO2_-SOCSR (9)

GWP_CO2_ is the carbon emissions from grain crop production per unit area (kgce/ha), and CO_2input_ is the carbon emissions from the upstream production and transport of agricultural inputs per unit area. FerN, FerP, FerK, Manu, Pest, Film, Dies, Elec and Straw represent nitrogen fertiliser, phosphate fertiliser, potassium fertiliser, manure, pesticide, film, diesel inputs, electricity inputs and the amount of straw burning, respectively. EF is the carbon emission factor corresponding to the upstream production of agricultural inputs and straw burning (EF_Burning_). N_2_O_soil_ is the emission of N_2_O from a given field induced by the application of nitrogen fertiliser and manure. EF_FerN-N2O_ is the nitrogen fertiliser-induced N_2_O emission factor, and EF_Manu-N2O_ is the manure-induced N_2_O emission factor. CH_4paddy_ is the CH_4_ emissions from rice paddies, and Growth-Days is the growth period of rice.

SOCSR is the carbon sequestered per unit area by grain crop production (kgce/ha); C_soc-FerN_ is the carbon sequestered per unit area through the application of nitrogen fertiliser; C_soc-straw_ is the carbon sequestered per unit area by returning straw; and C_soc-notil_ is the carbon sequestered per unit area by no-till practices.

**Appendix C *Factor selection for the carbon footprint model***

C1 *N_2_O emission factor for manure application*

The N_2_O emission factor for manure application was synthesised from the literature on field manure management in China and overseas prior to the beginning of 2014. Each study was selected using the following criteria: 1) the study was an experimental field trial and did not consider laboratory-based potted plant experiments; 2) the experiment included replicates, and the amount of nitrogen fertiliser applied was specified; 3) the studied crop types included the focal crops of the present study, namely, maize, wheat and rice; 4) the study provided information on the basic climate and soil physicochemical attributes analysed; and 5) since manure is often applied to the surface of fields in China, the study utilised surface application techniques for manure application. The N_2_O emission factor from manure application was calculated as the average result of the different experiments that were reviewed. N_2_O emissions included direct N_2_O emissions from the soil as well as indirect N_2_O emissions from NH_3_ volatilisation.

C2 *Soil SOC sequestration factor*

In this study, we used the empirical model for soil carbon sequestration proposed by F*ei et a*^l.45^ In the empirical model, 84 data pairs (28 test points) represent soil carbon sequestration associated with the application of fertiliser; 117 data pairs (45 test points) represent soil carbon sequestration associated with straw returning; and 19 data pairs (12 test points) represent soil carbon sequestration associated with no-till practices. This model effectively simulates the amount of soil carbon sequestration that occurs due to differences in the natural environment and the agricultural production measures (i.e., nitrogen fertiliser application, straw returning and no-till practices) utilised in the various primary wheat-, maize- and rice-producing areas in China.

C3 *Straw-burning carbon emission factor*

The carbon emission factor for straw burning used in this study was derived from the results discussed in Zha*ng et a*^l.4^, who used a custom-designed combustion and test device to simulate the open burning of straw in fields.In that study, rice straw was sampled in Shanghai, and wheat and maize straws were sampled in Hebei Province; the carbon emission factors of greenhouse gases, such as CO_2_ and NO_2_, were obtained.

**Appendix D *Crop division method***

In this study, we divided the main grain crop-producing areas of China by climatic zone and cropping system. First, China was divided into the following three main areas by the annual cumulative temperature: a tropical/subtropical zone, a warm temperate zone and a middle temperate zone. These areas were further divided into the following seven subareas: I (Guangxi and Hainan), II (Fujian, Jiangxi, Zhejiang, Hunan, Shanghai, Anhui (southern mountainous area), Hubei and Guangdong), III (Sichuan, Chongqing, Anhui (Jianghuai hilly area) and Jiangsu (southern and central areas)), IV (Beijing, Tianjin, Hebei, Henan, Shandong, Shaanxi (Xi’an, Xianyang and Weinan), Anhui (Huaibei Plain) and Jiangsu (northern area)), V (Jiangsu (Hexi Corridor and the alpine, humid irrigation area)), VI (Shanxi, most of Shaanxi, Gansu (arid area) and Xinjiang) and VII (Heilongjiang, Jilin, Liaoning and Inner Mongolia in the central and eastern parts of the county).

**Appendix E *Date source***

The survey was organised by the Research Group on Nutrient Management Strategy of the Centre for Resources, Environment and Food Security at China Agricultural University and began on May 12, 2014. Four of the 11 main agricultural counties were selected based on their grain yield and area, and in each county, the six main producing villages were selected based on household income level. In each village, eight samples were selected at random according to the roster of households to ensure the representativeness of the samples. A household survey was conducted, and a standard questionnaire form was completed by a researcher.

The survey respondents exhibited different levels of knowledge, different levels of understanding of agricultural operations and different degrees of cooperation with the survey; as a result, differences between the survey data and the actual situations were apparent. Therefore, the data had to be revised once the paper version of the questionnaire was converted to a digital format. The original questionnaire form was first checked to address input errors, and any remaining abnormal or missing values were verified by a follow-up telephone call and a household visit. Strict and complete data revision was conducted to ensure data quality and to guarantee the reliability of the statistical data analysis.
